# Targeting the non-neuronal cholinergic system in macrophages for the management of infectious diseases and cancer: challenge and promise

**DOI:** 10.1038/cddiscovery.2016.63

**Published:** 2016-10-17

**Authors:** Sandra Reichrath, Jörg Reichrath, Amira-Talaat Moussa, Carola Meier, Thomas Tschernig

**Affiliations:** 1 Department of Anatomy and Cell Biology, Saarland University, 66421 Homburg, Germany; 2 Department of Dermatology, Saarland University, 66421 Homburg, Germany

## Abstract

Macrophages represent key players of the immune system exerting highly effective defense mechanisms against microbial infections and cancer that include phagocytosis and programmed cell removal. Recent findings highlight the relevance of the non-neuronal cholinergic system for the regulation of macrophage function that opens promising new concepts for the treatment of infectious diseases and cancer. This mini review summarizes our present knowledge on this topic and gives an outlook on future developments.

## Highlights

- Key components of the non-neuronal cholinergic system (NNCS) including nAChR*α*7 are strongly expressed in macrophages.
- The NNCS regulates phagocytosis in macrophages via multiple signaling pathways including dynamin-2- and JAK2/STAT3-signaling pathways.
- The NNCS can suppress ‘don't eat me’ signals (interaction of CD47 and signal-regulatory protein alpha (SIRP*α*)) between macrophages and cancer cells.
- The NNCS can induce ‘eat me’ signals (translocation of calreticulin (CRT) from the endoplasmic reticulum (ER) to the cell surface) in target cells.
- Pharmacological modulation of the NNCS represents a promising new concept for cancer prevention and therapy.

## The relevance of the non-neuronal cholinergic system for macrophage function: old friends, revisited

### Macrophages – key players of the immune defense against microbial infections and cancer

**Figure 1:**
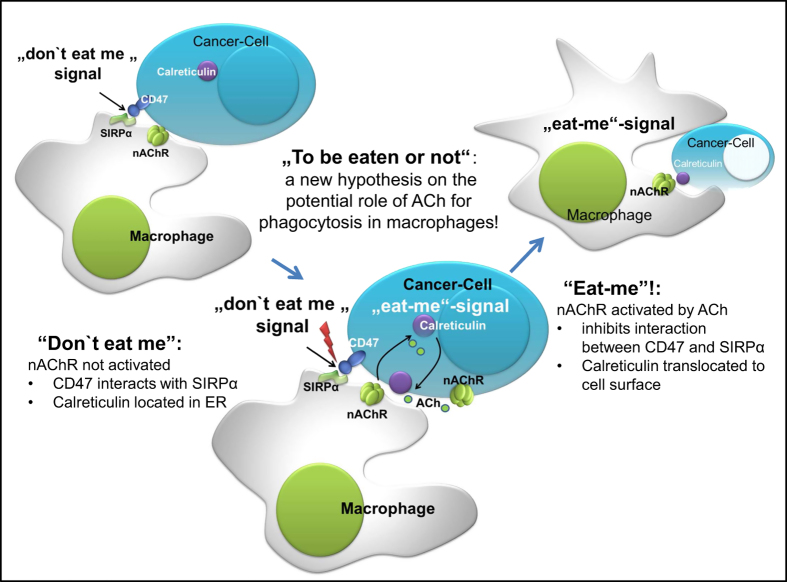
To be eaten or not: a new hypothesis on the potential role of ACh for macrophages/phagocytosis! Don't eat-me signal: cancer cells protect themselves against phagocytosis or programmed cell removal by overexpression of so-called don't eat-me signals such as CD47. Interaction of these don't eat-me molecules with SIRP*α* on the cell surface of macrophages protects cancer cells from phagocytosis. Eat-me signal: inhibition of the interaction between CD47 and SIRP*α*, and translocation of calreticulin from the ER to the cell surface act as eat-me signals and promote phagocytosis. Results of *in vitro* investigations and animal studies indicate that stimulation of nAChR by its ligand ACh may promote macrophage phagocytosis via inhibition of interaction between CD47 and SIRP*α,* and translocation of calreticulin from the ER to the cell surface. Moreover, nAChR activation may enhance endocytosis and phagocytosis via stimulated recruitment of GTPase dynamin-2 to the forming phagocytic plaque. ACh enhances bacterial uptake via inhibition of NF*κ*B activation and of pro-inflammatory cytokine production and stimulation of anti-inflammatory IL-10 production.

Macrophages are big, movable, eukaryotic cells, which develop from monocytes (blood cells which belong to the class of leukocytes) that have left the circulating blood, and that belong to the cellular immune system.^1^ One makes a distinction between mobile macrophages that immigrate need-wise from the blood into their varying target tissues, and locally restricted tissue-specific macrophages, which represent the large part of the macrophage population. Tissue-specific macrophages are bound to a distinct tissue and may differ greatly in their function and morphology (e.g., histiocytes in the connective tissue, microglia in the brain, alveolar macrophages in the lung, Kupffer cells in the liver, Hofbauer cells in the placenta, osteoclasts in the bones and Langerhans cells in the skin).^1^ As a component of the mononuclear phagocytic system, macrophages have varying functions within the scope of the cause defense, central role with the initiation and regulation of defense reactions (inflammation), with their most important mission being phagocytosis (Figure 1), and beyond it, destruction of tumor cells, elimination of cell detritus, antigen presentation and sore healing.^2^ In this context, it has to be emphasized that their most important job is phagocytosis (Figure 1) of microorganisms and other foreign bodies within the framework of the unspecific defense.^2^ The cytoplasm of the macrophage contains a large number of lysosomes that contain lytic enzymes, which contribute to the elimination of the pathogen. This process leads to the so-called ‘activation’ of the macrophage, and subsequently to the secretion of cytokines, which regulate the resulting inflammation process. In addition, macrophages have an important function as antigen-presenting cells that also involve phagocytosis.^1^ Macrophages process via phagocytosis pathogens to peptide fragments, which thereafter are presented with the help of major histocompatability complex (MHC) II molecules on the cell surface. These antigen–MHC-II complexes are then recognized by T-helper cells, resulting in specific immune reactions that may include the release of antibodies by B cells.

### The role of macrophages for cancer surveillance and elimination

Recently, it has been shown that macrophages can eliminate tumor cells via a highly regulated immunosurveillance mechanism called programmed cell removal (PrCR).^3^ However, cancer cells often express CD47 and/or other antiphagocytic ‘don't-eat-me’ signals that protect them from PrCR-mediated recognition and phagocytosis. Interestingly, blocking the interaction of CD47 on cancer cells and its corresponding receptor SIRP*α* on macrophages results in efficient PrCR of tumor cells but not of normal cells both *in vitro* and *in vivo*. Notably many cancer cells, in contrast to normal cells, express pro-phagocytic ‘eat-me’ signals such as CRT, that interact with other factors, including Bruton's tyrosine kinase (Btk), a member of the Tec non-receptor protein tyrosine kinase family, which has a crucial role in the regulation of the innate immune response. Recently it has been shown that macrophages express CRT, and that Btk-mediated toll-like receptor signaling results in trafficking of CRT to the cell surface, where this ‘eat-me’ signal can contribute to PrCR of cancer cells.^4^

### Acetylcholine – one of the most important neurotransmitters in humans

Acetylcholine (ACh), an ester of acetic acid and the monovalent aminoalcohol choline, represents the first neurotransmitter that has been identified.^5^ Otto Loewi proved in 1921 in a frog heart that a chemical, which he called first vagus compound, and which Henry H Dale identified later as ACh, was responsible for the transfer of a nervous impulse to the heart.^5^ Today, ACh is known as one of the most important neurotransmitters in humans and many other organisms.^5–7^ ACh is regarded as a classical neurotransmitter that exerts its effects at least in part via binding to two different classes of receptor molecules, the nicotinic (nAChR; that are also stimulated by nicotine) and the muscarinic ACh receptors (mAChR; that are also stimulated by the mushroom poison muscarin), that are both expressed in different subtypes.^7^ nAChRs have been characterized as binding and effector proteins to mediate chemical neurotransmission at neurons, ganglia, interneurons and the motor endplate.^8^ mAChRs have been characterized as binding and effector proteins to mediate chemical neurotransmission at neurons and effector organs such as heart, smooth muscle fibers and glands.^8^ This historical view of ACh acting exclusively as a neurotransmitter has recently been revised based on observations published both early and late in the last century, identifying the NNCS.^8^

### The non-neuronal cholinergic system – unraveling hidden secrets

The NNCS represents an ancient regulatory network that already existed from the beginning of life in non-neuronal cells like bacteria, algae and protozoa long before the neural system had developed (reviewed in^5–8^). The ubiquitous synthesis of ACh and the expression of n- and mAChRs in mammalian cells gives an impressive example of the complexity of biological systems.^8^ All receptor subtypes and signal transduction pathways used by cholinergic neurons are also used by single non-neuronal cells to communicate among each other and to maintain their phenotypic functions and thus organ homoeostasis.^8^ Up to now, little is known about the relevance of the NNCS for human health, but recent findings highlight its importance for the regulation of macrophage function, opening promising new concepts for the treatment of infectious diseases and cancer^9,10^ (Table 1).

### The relevance of the non-neuronal cholinergic system for regulation of macrophage function

As outlined above, macrophages represent key players of the immune system exerting highly effective defense mechanisms against microbial infections and tumor cells that include phagocytosis and PrCR. Recent findings highlight the relevance of the NNCS for the regulation of macrophage function and open promising new concepts for the treatment of infectious diseases and cancer. In monocytes, monocyte-derived macrophages, lung and alveolar macrophages from non-smokers, smokers and COPD patients, expression of the high-affinity choline transporter, choline acetyltransferase, vesicular ACh transporter and muscarinic receptors (M(1)–M(5)) were demonstrated using real-time PCR (Table 1).^11^ In that study, M(2) and M(3) receptor expression was confirmed using immunocytochemistry.^11^ Interestingly, an important role of nAChR*α*7 expression and function in macrophage survival and apoptosis using *in vitro* polarized (M1 and M2) bone marrow-derived macrophages (BMDMs) from wild-type and nAChR*α*7 knockout mice has been demonstrated (Table 1).^12^ These findings show that stimulation of nAChR*α*7 results in activation of the STAT3 pro-survival pathway and protection of macrophages from ER stress-induced apoptosis. These actions are rather selective for M2-BMDMs and are associated to activation of the JAK2/STAT3 axis. Remarkably, these effects are completely lost in M2 macrophages lacking nAChR*α*7. Recently, it was shown that *α*7nAChR represents a promising pharmacological target to improve the clinical outcome of patients on ventilators by augmenting host defense against bacterial infections.^13^ Hyperoxia is routinely used to treat patients with respiratory distress. However, prolonged exposure to hyperoxia compromises the ability of the macrophage to phagocytose and clear bacteria. It was shown that the exposure of mice to hyperoxia elicits the release of the nuclear protein high-mobility group box-1 (HMGB1) into the airways. Extracellular HMGB1 impairs macrophage phagocytosis and increases the mortality of mice infected with *Pseudomonas aeruginosa*. Recently, it was demonstrated that GTS-21 [3-(2,4 dimethoxybenzylidene)-anabaseine dihydrochloride], an *α*7nAChR agonist, inhibits hyperoxia-induced HMGB1 release into the airways, enhances dose-dependently phagocytic activity of macrophages and improves bacterial clearance from the lungs in a mouse model of ventilator-associated pneumonia. Moreover, it was shown that vagus nerve activity augments intestinal macrophage phagocytosis via nAChR*α*4*β*2 (Table 1).^14^ The vagus nerve negatively regulates macrophage cytokine production via the release of ACh and activation of nAChRs. In various models of intestinal inflammation, vagus nerve efferent stimulation ameliorates disease. Using fluorescence-activated cell sorter analysis, short-hairpin RNA-assisted gene knockdown, and the use of specific nAChR knockout mice, it was shown that nAChR activation enhanced endocytosis and phagocytosis in macrophages residing in the peritoneal and mucosal compartments (Table 1).^14^ This effect was mediated via stimulated recruitment of GTPase dynamin-2 to the forming phagocytic cup.^14^ These effects involve nAChR*α*4/*β*2, rather than nAChR*α*7. Despite enhanced bacterial uptake, ACh reduced NF-*κ*B activation and pro-inflammatory cytokine production, while stimulating anti-inflammatory interleukin-10 production. Vagus nerve stimulation in mice altered mucosal immune responses by augmenting epithelial transport and uptake of luminal bacteria by lamina propria macrophages.^14^ ACh enhances phagocytic potential while inhibiting immune reactivity via nAChR*α*4/*β*2 in mouse macrophages. Hence, vagus nerve efferent activity may stimulate surveillance in the intestinal mucosa and peritoneal compartment.^14^

Recently, Kyoto Encyclopedia of Genes and Genomes (KEGG) pathway analysis of differentially regulated miRNAs in whole-rat brains (that contain microglia) after exposure of animals with lipopolysaccharide, Ab*α*7 (directed against AChR*α*7) or nicotine revealed regulation of various signaling pathways that are involved in phagocytosis, including Fc gamma R-mediated phagocytosis, Jak-STAT-, PI3K-Akt-, Calcium-, mTOR-, Notch- and Wnt-signaling pathways.^15^

### Conclusions and outlook

Macrophages represent key players of the immune system exerting highly effective defense mechanisms against microbial infections and cancer, which include phagocytosis and PrCR. Recent findings highlight the relevance of the NNCS for the regulation of macrophage function that opens promising new concepts for the treatment of infectious diseases and cancer.^14,16–19^

Until today, many compounds have been identified, which directly or indirectly can increase the effects of ACh on corresponding receptor molecules. The different cholinesterase inhibitors (actually, ACh inhibitors) belong to the latter. Irreversible inhibitors of the cholinesterase include well-known insecticides such as parathion (E 605), or other highly neurotoxic chemical agents such as sarin, tabun and many others, which often even in small quantities cause death by overstimulation of cholinergic synapses. Other chemical compounds such as neostigmin belong to the group of reversible cholinesterase inhibitors. Parathion is also used as an antidote for curare. The antagonistic interplay between parathion and curare gives an impressive example of the complexity of the NNCS, and highlights the diversity of its multiple promising targets for pharmacological modulation that may also open new avenues for the pharmacological regulation of macrophage function. Curare blocks the docking station for ACh, thereby immobilizing the nervous system and causing suffocation death. Because the active substances of curare are competitive tailors, their effect can be antagonized by an excess of ACh. Blocking of acetylcholinesterase by parathion results in such an excess of ACh and in restoring nerve cell function. However, parathion is also a poison, and aftercare with atropin is often necessary. Another interesting chemical that modulates the NNCS is physostigmin (eserin) that prevents the splitting of ACh by cholinesterase in choline and acetate. Moreover, a broad variety of compounds has been identified that block the effects of ACh on its corresponding receptors (most importantly muscarinic receptors), exerting the so-called anticholinergic effect. Certain alkaloids work anticholinergic, for example, atropin, hyoscyamin or scopolamin.

It has to be emphasized that the NNCS interacts with a broad variety of other hormonal networks including the adrenergic system. As adrenergic receptors are also expressed in macrophages^20^ and the adrenergic system is a direct opponent of the cholinergic system in the nervous system,^21^ it is tempting to speculate whether these two systems antagonize each other in macrophages as well.

In conclusion, an increasing body of evidence now convincingly demonstrates that pharmacological modulation of the NNCS to regulate phagocytosis and PrCR in macrophages represents a promising new strategy for the prevention and/or therapy of many health disorders including infectious diseases and various types of cancer.

## Figures and Tables

**Table 1:** Targeting phagocytosis: the non-neuronal cholinergic system in macrophages

*Target*	*Function*	*Relevance for targeting phagocytosis*
*Nicotinic ACh receptors*		
*α*7nAChR	Enhances phagocytic activity, improves bacterial clearance from the lungs, activates the STAT3 pro-survival pathway and protects from ER stress-induced apoptosis	Promising target for pharmacological modulation of phagocytosis and apoptosis in macrophages
*α*4/*β*2 nAChR	Enhances endocytosis and phagocytosis in macrophages residing in the peritoneal and mucosal compartments stimulate recruitment of GTPase dynamin-2 to the forming phagocytic cup	Promising target for pharmacological modulation of phagocytosis and endocytosis
		
*Muscarinic ACh receptors*		
M1–M5 ACh receptors	Release of LTB4 (M3)	Potential target for pharmacological modulation of phagocytosis
*ACh*	Varying effects on phagocytosis depending on the presence of corresponding receptors	Potential target for pharmacological modulation of phagocytosis by modulating its synthesis/metabolism or by using agonists/antagonists
